# Supplementary material for: Extensive variation and strain-specificity in dengue virus susceptibility among African Aedes aegypti populations
Source: PLoS Negl Trop Dis. 2024 Mar 25;18(3):e0011862. doi: 10.1371/journal.pntd.0011862 (PMC10994562; doi:10.1371/journal.pntd.0011862)
Supplement: Attachment — Submitted filename: Dabo_response_to_reviewers.docx [file pntd.0011862.s003.docx]

**Response to editor and reviewers**

**Editor comments to authors:**

The reviewers tended to like the study and had some minor improvements. Specifically, I tend to agree with Reviewer 2 that titer data should be calculated by removing the zero values. The proportion data tells us how many became infected whereas the titer data should tell us how well the virus is able to replicate in those mosquitoes that became infected. Also, Reviewer 1 suggested adding some caveats to the discussion about the potential that inbreeding of the colonies and passaging of the virus could have on the outcomes. Reviewer 3 pointed out some useful citations concerning DENV activity in Africa. Some text should be added to the manuscript regarding DENV activity in Africa. Reviewer 3 also questioned the conclusions about transmission potential of your disseminated mosquitoes. Additional studies are not required to address this but justifying your conclusions with a reference(s) would be recommended.

*Response* : We appreciate the constructive feedback and the reviewers’ suggestions to improve the paper. We re-analyzed dissemination prevalence and non-zero dissemination titers separately. We added a new paragraph to discuss the limitations of the study related to the relatively high number of laboratory generations of our field-derived mosquito colonies. We expanded our discussion on the records of DENV outbreaks in Africa. Our detailed point-by-point responses are provided below. Lines numbers refer to the version of the manuscript with tracked changes.

**Reviewer's Responses to Questions**

Key Review Criteria Required for Acceptance ?

As you describe the new analyses required for acceptance, please consider the following:

Methods

-Are the objectives of the study clearly articulated with a clear testable hypothesis stated?

-Is the study design appropriate to address the stated objectives?

-Is the population clearly described and appropriate for the hypothesis being tested?

-Is the sample size sufficient to ensure adequate power to address the hypothesis being tested?

-Were correct statistical analysis used to support conclusions?

-Are there concerns about ethical or regulatory requirements being met?

Reviewer #1: Below I pointed the issues related to mosquito lines and inbreeding and DENV isolates (time) which could have impacted the results.

*Response* : We addressed the questions on the passage history of virus strains and mosquito colonies below.

Reviewer #2: Yes

Reviewer #3: The objectives of the study are clearly articulated, however, a few areas need to be clarified or discussed:

What were the individual DENV blood meal titers used to infect each mosquito population in order to come up with the OID50?

*Response* : All the blood meal titers are indicated in Figure 1 (x-axis). The OID_50_ values are estimated from the logistic regression of the dose-response curves. We added this information on lines 146-147.

Why were 2 different methods or approaches used to determine the infection and dissemination rates? That is PCR to determine infection rates and FFA to determine the dissemination rates.

*Response* : We used different methods because they served a different purpose. We used RT-PCR as a reliable and sensitive assay to determine infection rate. We used FFA to quantify infectious virus in head tissues because dissemination titer is a proxy for DENV transmission potential (Lambrechts *et al. J Virol* 2012). We added this information on lines 347-349.

--------------------

Results

-Does the analysis presented match the analysis plan?

-Are the results clearly and completely presented?

-Are the figures (Tables, Images) of sufficient quality for clarity?

Reviewer #1: Yes

Reviewer #2: Yes

Reviewer #3: To create clarity and allow the reader to interpret the results better, other than the graphs, it will be important to provide a table that provides raw figures of the mosquitoes exposed to each viral titer, the percentage infected and the percentage disseminating the virus.

*Response* : The raw data are provided in Table S1. We added two tabs with the requested information for both infection and dissemination (number of mosquitoes tested, number of mosquitoes positive, proportion) for each virus-population-dose combination.

It may be an overreach to state that dissemination can be considered as transmission potential as many studies have shown high levels of dissemination into the body but lack of transmission of the virus in expectorated saliva. It may be only safe to consider pathogens present in the salivary gland as that likely to be transmitted to the saliva.

*Response* : We agree with the Reviewer that dissemination does not equate transmission potential. On lines 179-180, we refer to an earlier study showing that DENV dissemination titer is a strong predictor of infectious virus presence in saliva (Lambrechts *et al. J Virol* 2012).

It may be important to explain why the Guadaloupe reference strain was excluded during the dissemination analysis

*Response* : We excluded the Guadeloupe strain because we were primarily interested in the variation among African populations, not in the expected difference between African populations and the non-African reference. We added this information on lines 139-140.

Line 174 and 175 should be altered to demonstrate what you're study investigated as you investigated dissemination but not transmission.

*Response* : We modified this sentence to restrict our conclusion to virus dissemination (line 183).

--------------------

Conclusions

-Are the conclusions supported by the data presented?

-Are the limitations of analysis clearly described?

-Do the authors discuss how these data can be helpful to advance our understanding of the topic under study?

-Is public health relevance addressed?

Reviewer #1: Partially

Reviewer #2: Yes

Reviewer #3: It will be important to include in your discussion the effect of using a low lab generation of Cape Verde on the overall results.

*Response* : To address this point, we analyzed the OID_50_ estimates as a function of the number of laboratory generations of each mosquito colony (averaged across the study period). For all the DENV strains, we found no effect of the number of laboratory generations on the OID_50_ estimates (linear regressions: R^2^=0.01–0.51; *p*=0.175–0.828). We added this information on lines 171-172.

Lines 179-180: This might need to be re-worded as there have been contradictory findings on the vector competence of Aaf for DENV and YFV

*Response* : We apologize if this is a misunderstanding, but this is precisely the conclusion of the present study. We show that for multiple DENV strains, *Aaf* populations are not always less susceptible than their *Aaa* counterparts.

There have also been reports of several Dengue outbreaks in African countries driven by local populations of Aedes aegypti, hence there is a possibility that African populations of Aedes aegypti are susceptible to Dengue virus

Dickson, L. B., Sanchez-Vargas, I., Sylla, M., Fleming, K. & Black, W. C., 4th. Vector competence in West African Aedes aegypti Is Flavivirus species and genotype dependent. PLoS Negl. Trop. Dis. 8, e3153 (2014).

Tabachnick, W. J. et al. Oral infection of Aedes aegypti with yellow fever virus: geographic variation and genetic considerations. Am. J. Trop. Med. Hyg. 34, 1219–1224 (1985).

Bosio, C. F., Beaty, B. J. & Black, W. C., 4th. Quantitative genetics of vector competence for dengue-2 virus in Aedes aegypti. Am. J. Trop. Med. Hyg. 59, 965–970 (1998).

*Response* : Again, we entirely agree with the Reviewer that some African populations of *Ae. aegypti* are susceptible to DENV since this is what the present study demonstrates. We further argue that variation in DENV susceptibility among African *Ae. aegypti* populations may contribute to explain the observed heterogeneity in dengue incidence in Africa on lines 267-270.

Lines 216-217 may need to be re-worded

There have been reports of several Dengue outbreaks in African countries that have been documented in the past: This is just a single country:

Johnson, B. K. et al. Epidemic dengue fever caused by dengue type 2 virus in Kenya: preliminary results of human virological and serological studies. East Afr. Med. J. 59, 781–784 (1982).

Konongoi, L. et al. Detection of dengue virus serotypes 1, 2 and 3 in selected regions of Kenya: 2011-2014. Virol. J. 13, 182 (2016).

Gathii, K., Nyataya, J. N., Mutai, B. K., Awinda, G. & Waitumbi, J. N. Complete Coding Sequences of Dengue Virus Type 2 Strains from Febrile Patients Seen in Malindi District Hospital, Kenya, during the 2017 Dengue Fever Outbreak. Genome Announc. 6, (2018).

Muthanje, E. M. et al. March 2019 dengue fever outbreak at the Kenyan south coast involving dengue virus serotype 3, genotypes III and V. PLOS Global Public Health vol. 2 e0000122 Preprint at [https://urldefense.com/v3/__https://doi.org/10.1371/journal.pgph.0000122__;!!JFdNOqOXpB6UZW0!tekXap6zeBwpF1CoR8wPbrSHMcCyz54tJ8pk_VkuCnZGYZl51VQ-Z1MXmTlUKS_HGbC8aXO7NGyGjo06aniUbnW8$](https://urldefense.com/v3/__https:/doi.org/10.1371/journal.pgph.0000122__;!!JFdNOqOXpB6UZW0!tekXap6zeBwpF1CoR8wPbrSHMcCyz54tJ8pk_VkuCnZGYZl51VQ-Z1MXmTlUKS_HGbC8aXO7NGyGjo06aniUbnW8$)

*Response* : We thank the Reviewer for these additional references. We expanded our discussion on the records of DENV outbreaks in Africa, including 6 new references, on lines 261-263.

--------------------

Summary and General Comments

Use  this section to provide overall comments, discuss strengths/weaknesses of the study, novelty, significance, general execution and scholarship. You may also include additional comments for the author, including concerns about dual publication, research ethics, or publication ethics. If requesting major revision, please articulate the new experiments that are needed.

Reviewer #1: The present study performed by Dabo and colleagues studied the vector competence of African Aedes aegypti populations towards DENV isolated from Africa. The manuscript is well written, and the methods, although simplified, were appropriate for the topic in question. The work is quite simple and had no major link around the vector competence of African mosquito populations and DENV, maybe because of the points discussed below.

Major points

Mosquito populations: 5-25 generations in the lab means a lot of inbreeding and might not represent the field component of the mosquito line and origin.

*Response* : We agree with the Reviewer that the relatively high number of laboratory generations of our field-derived mosquito colonies is a limitation of the study. We added a new paragraph in the discussion on lines 285-290 to address this important point.

Virus isolates: Isolates have been collected quite long ago and multiple passaging could impact on the infectivity. It was not present on the methods section how these isolates have been kept (whether in constant passaging, which could impact mosquito infectivity; or as frozen stocks). Also, it was a pity that there were no respective virus isolates to match each mosquito population. Most of the isolates came from Gabon.

Including a discussion around these caveats would be beneficial to the study.

*Response* : The virus strains only underwent 3 to 10 passages in mosquito cells prior to their use in the study. We added this information in Table 2. We did not attempt to match the geographic origin of the viruses with that of the mosquitoes because local adaptation was not the focus of the study. In an earlier study, we find no evidence of DENV local adaptation to *Ae. aegypti* populations (Fansiri *et al. Evol Appl* 2016).

Reviewer #2: The manuscript by Dabo et al. aims to measure whether African populations of Ae. aegypti are more refractory to DENV compared to non-African populations. The authors compare the vector competence of panel of Ae. aegypti lines from within Africa to a reference colony collected outside Africa. Using a panel of different DENV serotypes and genotypes the authors clearly demonstrates that unlike with ZIKV, the vector competence of the African lines is not lower than a line from Guadeloupe in all cases. Instead, vector competence depends on the specific pairing between mosquito population and DENV strain. The manuscript is very well written and the data clearly supports the conclusions.

Minor Comments:

Line 45: Seven colonies is hardly a continent-wide survey. Language should be softened.

*Response* : We removed the term “continent-wide”.

Figure 3: This figure is hard to read. Zeroes should not be included when comparing the titers between individuals. It is more informative to compare titers of those individuals who are infected and then separately plot the percentage of individuals with a disseminated infection.

*Response* : We re-analyzed dissemination titers separately for dissemination prevalence and non-zero dissemination titers. The corresponding figures (Fig. 3 and Fig. 4) and tables (Table 4 and Table 5) were updated accordingly.

Reviewer #3: The authors may need to provide a bit more clarity with regard to their data presentation as well as update and highlight other findings and publications about Dengue circulation and outbreaks in several countries in Africa which may explain some of their findings. This will bring clarity and improve their discussion sections. Comments on specific sections have been provided above.

*Response* : We expanded our discussion on the records of DENV outbreaks in Africa, including 6 additional references, on lines 285-290.
